# Psychometric properties of the Persian version of Instrument of Professional Attitude for Student Nurses (IPASN)

**DOI:** 10.1002/nop2.682

**Published:** 2020-11-20

**Authors:** Mohammad Eghbali, Razieh Bandari, Majideh Heravi‐Karimooi, Fariba Ghaesemzadeh, Ali Montazeri

**Affiliations:** ^1^ Student Research Committee University Of Social Welfare and Rehabilitation Sciences Tehran Iran; ^2^ Social Determinants of Health Research Center Semnan University of Medical Sciences Semnan Iran; ^3^ Elderly Care Research Center, College of Nursing & Midwifery Shahed University Tehran Iran; ^4^ Kowsar Hospital Semnan University of Medical Sciences Semnan Iran; ^5^ Population Health Research Group, Health Metrics Research Centre Iranian Institute for Health Sciences Research, ACECR Tehran Iran; ^6^ Faculty of Humanity Sciences University of Science &Culture, ACECR Tehran Iran

**Keywords:** internal consistency, nursing students, professionalization questionnaire, validity

## Abstract

**Aim:**

To evaluate nursing students’ professionalism attitude, it is necessary to use a validated and reliable instrument.

**Design:**

This study aimed to develop the Persian version of the eight‐dimensional professionalism attitude questionnaire for student nurses (Instrument of Professional Attitude for Student Nurses).

**Methods:**

In this cross‐sectional study during March to April 2017, after being translated into Persian and receiving experts’ comments face, content, and construct validity of the questionnaire were sought among nursing students. Internal consistency reliability was examined by estimating the Cronbach's alpha coefficient and stability was estimated by calculating intraclass correlation coefficinet.

**Results:**

A total of 750 students with an average age of 24.15 (*SD =* 1.32) years participated in the study. Both exploratory and confirmatory factor analyses confirmed the construct of the questionnaire. Cronbach's alpha coefficient for the entire instrument was 0.89 and more than 0.70 for all dimensions.

Also the intraclass correlation coefficient for the questionnaire was found to be 0.92, well above acceptable threshold. The findings approved that the Iranian version of Instrument of Professional Attitude for Student Nurses is a reliable and valid questionnaire. Hence, using this questionnaire to conduct studies is recommended.

## 1 INTRODUCTION

Nursing has changed over the past three decades. For instance, the development of the roles and independence in medical clinics has brought about educational standards and licensing for nurses. At present, there are a growing number of nurses with different specialty in medical clinics (Salmond & Echevarria, 2017). Thus, professionalization has become important issue for nursing practice. Professionalization is defined as the degree of commitment to values and behavioural characteristics of a specific profession (Shohani & Zamanzadeh, 2017). Although professionalization is considered as a framework for determining a single specific kind of job among a social community, the emphasis on commitment to the values might led towards an understanding of a specific work condition (Ghadirian et al., 2014). Throughout the years, several nursing investigators have defined nursing characteristics. Based on the previous studies, encouraging nursing students to acquire professional attitudes and behaviours is believed to be of great importance (Hisar & Karadağ, 2010; Karadağ et al., 2007).

## 2 BACKGROUND

Professional attitudes in nursing students might develop and form during their studies right before starting their professional services (de Swardt et al., 2017). In this context, one of the most fundamental goals of nursing education should be to teach the students the area of professional identity (Heshmati Nabavi et al., 2014). As such, it is necessary to plan for nursing education in all institutions in a way to develop professional attitudes. In doing so, the capacity for changing the attitude in nursing educational institutes needs to be analysed (Van Graan et al., 2016). On the other hand, one of the most noticeable reasons for studying nurses’ attitudes before they begin their services is to find some clues on attitudes and behaviours of those students who will teach in their future professional life. This could be sought by determining the students’ attitudes prior to their professional life and to help them realize any potential problems and pitfalls in the future (Karadağ et al., 2016).

Although a variety of instruments exist to assess nurses’ professional attitudes and behaviours (Akhund et al., 2014; Aramesh et al., 2009; Arnold et al., 1998; Blackall et al., 2007; Blue et al., 2009; Bustamante & Sanabria, 2014; Crossley & Vivekananda‐Schmidt, 2009; DeLisa et al., 2001; Hisar et al., 2010; Jiang et al., 2010; Lombarts et al., 2014; Miller et al., 1993; Nhan et al., 2014; Tsai et al., 2007; Wittich et al., 2013), the only specific instrument that evaluates nursing students’ attitudes and behaviours regarding professionalism is the one that was developed by Hisar et al. (2010) (Çelik et al., 2012).

Hisar and her colleagues designed a list for nursing students’ professionalism attitude namely the Instrument of Professional Attitude for Student Nurses (IPASN). The original questionnaire is in Turkish, and the English version of the questionnaire extensively was used by many investigators (Courtney‐Pratt et al., 2015; Heshmati Nabavi et al., 2014; A. Karadağ et al., 2015; Karadağ et al., 2016; Xiao et al., 2017; Çelik & Hisar, 2012). The only version of the questionnaire in other languages is the Chinese version, and the psychometric properties of the questionnaire are well documented (Karadağ et al., 2016).

Due to the importance of recognition and evaluation of professionalism attitude among nursing students, this study aimed to translate and validate the questionnaire in Iran. Currently, such a questionnaire is not available in Iran. It was hoped this might help to improve nursing discipline in higher education and in practice.

## 3 METHODS

### 3.1 The questionnaire

The Instrument of Professional Attitude for Student Nurses (IPASN) includes 28 items rating on a 5‐point scale tapping into eight dimensions: Contribution to the increase of scientific information load (6 items); Autonomy (3 items); Cooperation (5 items); Competence, continuous education (3 items); Participation in professional organizations and professional development (3 items); Working in committees (3 items); Community service (2 items); and Ethical codes and theory (3 items) (Hisar et al., 2010; Çelik et al., 2012).

### 3.2 Procedure and translation

The present study was a methodological and validation study. After obtaining permission, the translation process from English into Persian was conducted using the forward–backward method. First, the items were translated into the target language by two experts who had a good command of both languages. Then, two translators re‐translated the materials into the original language, that is English. The translated items were matched with the original items to ensure that the concepts had been successfully conveyed. Afterwards, to check the content and face validity of the instrument, the questionnaires were distributed among five Persian language and literature experts to get their suggestions regarding language‐related revisions, use of words and the appropriateness of the items and their placement. Then, the first draft of the Persian version of the questionnaire was pre‐tested on ten senior nursing students and the face validity and item combination were then analysed. Additionally, qualitative content validity was examined by a panel of experts. After applying the required revisions, the final version of the Persian questionnaire was developed and was administered to a sample of nursing students.

### 3.3 Sample and setting

A multistage stratified sampling procedure was applied to include a representative sample of nursing students in the study. As such, 15 nursing colleges from the whole country (five main regions of the country: north, south, east, west and centre) were randomly selected. Then in each college, a convenient sample of senior nursing students who were willing to participate were entered into the study. For the study purposes, we thought at least 280 students (10 students per item) are needed for exploratory factor analysis (EFA) and similarly 280 students are needed for confirmatory factor analysis (CFA) (Polit & Yang, 2015). However, in practice we recruited 750 students (450 for EFA and 300 for CFA).

### 3.4 Data collection

The data were collected during March ‐ April 2017. The interviews were conducted in the corresponding hospitals relevant to the above universities during the internship period. The demographic information including age, sex and marital status also was recorded.

### 3.5 Statistical analysis

1. Validity: Validity of the questionnaire was tested using both exploratory and confirmatory factor analyses.
  - The exploratory factor analysis was performed to explore the factor structure the questionnaire. The Kaiser–Meyer–Olkin (KMO) and Bartlett's test (BT) were performed for sampling adequacy and sphericity of the data. Eigenvalues above 1 and scree plot were used to determine the number of factors. When the KMO index is higher than 0.60 and Bartlett sphericity test results are significant, it means that the data can be analysed using factor analysis. Loading of 0.3 or above considered acceptable to maintain each item in an extracted factor.
  - The confirmatory factor analysis (CFA) was performed to confirm the factor structure of the IPASN. The CFA model was tested using maximum likelihood estimates. The goodness of fit of the model was appraised using multiple criteria including the followings: Root Mean Square Error of Approximation (RMSEA); Goodness of Fit Index (GFI); Adjusted Goodness of Fit Index (AGFI); Non‐normed Fit Index (NNFI); Normed Fit Index (NFI); Incremental Fit Index (IFI); and Comparative Fit Index (CFI). The Lisrel was used for the CFA. AGFI: Adjusted Goodness of Fit Index.
2. Reliability: To determine the internal consistency and stability, Cronbach's alpha coefficient and intraclass correlation coefficient (ICC) for the whole scale were calculated (Rejeh et al., 2012). Statistical analysis was provided using SPSS version 16 and LISREL 8. The maximum acceptable type 1 error was considered as 5%.

## 4 RESULTS

### 4.1 The study sample

All 750 nursing students were entered into the study. Of these, data from 450 students were used for exploratory factors analysis and the data from the remaining 300 students were used for confirmatory factor analysis. The characteristics of the study samples are shown in Table 1. The mean age of participants was 23.34 (*SD* 1.11) years, and more than half (61.86%) were female students. Most participants were single (60.40%). Table 1 presents further information of the study participants.

**TABLE 1:** Demographic characteristics of nursing students

Characteristics				
Age	Mean (*SD*)		23.34 (1.53)	24.15 (1.32)
		(*N* = 750)	(*N* = 450)	(*N* = 300)
Gender	Male	286 (38.14)	219 (48.70)	67 (22.34)
	Female	464 (61.86)	231 (51.30)	233 (77.66)
Marital status	Single	453 (60.40)	266 (59.10)	187 (62.34)
	Married	297 (39.60)	184 (40.90)	113 (37.66)

### 4.2 Exploratory factor analysis

The KMO value for the data was 0.877, and the results obtained from the Bartlett's sphericity test were satisfactory (*p* < .0001; χ^2^ = 4,739.927) suggesting that the sample was adequate and the distribution of the data was acceptable for exploratory factor analysis. Consequently, factor analysis was conducted and 8 factors with eigenvalue equal or greater than 1 extracted that jointly explained 73.06% of the variance observed. The factor loading ranged from 0.57–0.93. The results are shown in Table 2.

**TABLE 2:** The results obtained from factor analysis of twenty‐eight items of the instrument (Extraction: Principal Component; Rotation: Promax; F: Factor^a^)

Items	F1	F2	F3	F4	F5	F6	F7	F8
1. I want to do a Master's in nursing	**0.797**	0.017	0.160	0.100	0.206	0.109	0.214	0.135
2. I want to do PhD work in nursing	**0.871**	0.002	−0.047	0.134	0.178	0.165	0.178	0.168
3. After graduation, I want to write articles on my field of interest	**0.857**	−0.071	0.128	0.160	0.076	0.339	−0.053	0.212
4. I want to develop skills and information in research	**0.747**	0.018	0.225	0.101	0.241	0.218	−0.055	0.144
5. I want to participate in investigations on health in nursing	**0.809**	−0.004	0.103	−0.163	0.084	0.132	0.357	0.104
6. I want to work in research	**0.779**	0.124	0.077	0.088	0.030	0.142	0.076	0.339
23. Nursing services should be managed by nurses	0.339	**0.822**	0.199	0.051	0.214	0.146	0.241	0.218
24. Nurses must be included in hospital boards	0.218	**0.790**	0.271	−0.150	0.084	0.217	0.120	0.258
25. Nurses make the decisions on nursing care	0.258	**0.739**	0.213	−0.017	0.036	0.194	0.059	0.084
7. I do not plan to conduct projects	0.084	0.132	**0.826**	−0.053	0.134	0.146	0.095	0.030
10 I do not plan to be a member of international organizations regarding my field	0.030	0.142	**0.764**	0.189	0.078	0.168	0.079	0.185
11. I do not plan to be a member of health organizations other than nursing	0.094	0.153	**0.744**	0.004	0.248	0.160	0.012	0.176
14. I do not plan to participate in studies aiming to protect the social rights of the community	0.087	0.100	**0.600**	0.077	0.149	0.154	0.095	0.046
19. I do not plan to use the ethic codes of ICN in my practice	−0.021	0.211	**0.745**	0.127	0.160	−0.053	0.017	0.160
16. I think I should have a certificate awarded by Ministry of Health in my area	0.382	0.124	0.017	**0.795**	0.101	−0.055	0.002	−0.047
17. I think I should have a certificate awarded by universities or professional organizations (course document) in my area	0.376	0.187	0.086	**0.871**	−0.163	0.357	−0.071	0.128
18. I think I should have certificates awarded by other relevant institutions (hosp, etc.) in my area	0.371	0.167	0.095	**0.803**	0.088	0.076	0.018	0.048
8. I plan to be a member of the Iranian Nurses' Association	0.360	0.186	0.002	0.017	**0.714**	0.241	−0.004	0.051
9. I plan to be a member of special branch associations in my area	0.354	0.155	0.123	0.002	**0.738**	0.036	0.120	−0.150
15. I plan to subscribe to publications (journal etc.) on nursing	0.135	0.038	0.222	−0.071	**0.939**	0.146	0.059	−0.017
26. I want to take part in ethics committees	0.168	0.178	0.164	0.018	0.225	**0.939**	0.095	0.149
27. I want to take part in studies on quality control	0.212	0.149	0.147	−0.004	0.103	**0.713**	0.079	0.165
28. I want to serve as consultant/counsellor on areas related to nursing	0.144	0.203	0.102	0.124	0.077	**0.704**	0.012	0.192
12. I plan to work in organizations working for the benefit of community	0.104	0.205	0.154	0.216	0.077	0.265	**0.573**	0.095
13. I plan to participate in studies aiming to protect the health of the community	0.190	0.230	0.178	0.166	0.012	0.133	**0.701**	0.122
20. I think I should have copy of national ethical codes	0.113	0.151	0.179	0.178	0.146	0.198	0.015	**0.616**
21. I utilize nursing models in planning nursing care	0.248	0.135	0.178	0.002	0.165	0.194	0.056	**0.895**
22. I utilize the theories of disciplines other than nursing in planning nursing care	0.272	0.254	0.168	0.216	0.249	0.199	0.177	**0.787**
**Eigenvalue**	13.442	13.027	12.501	10.741	9.691	5.275	4.451	3.942
**% variance**	3.764	3.647	3.500	3.007	2.714	1.477	1.246	1.104
**Cronbach's alpha**	0.870	0.977	0.959	0.708	0.733	0.710	0.788	0.935
**ICC**	0.950	0.980	0.970	0.810	0.840	0.815	0.879	0.955

Bold values are acceptable factor loadings for the given factor as follows F1: Contribution to the increase of scientific information load, F2: Autonomy, F3: Cooperation, F4: Competence, continuous education, F5: Participation in professional organizations and professional development, F6: Working in committees, F7: Community service, F8: Ethical codes and theory.

### 4.3 Confirmatory factor analytic

**FIGURE 1:**
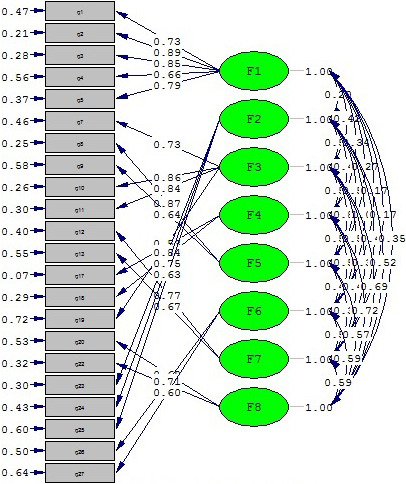
Results of the CFA: the standardized coefficients for the IPASN

According to the CFA, the multiple criteria obtained for the data were as follows: RMSEA = 0.07; GFI = 0.90; AGFI = 0.88; NNFI = 0.91; NFI = 0.91; IFI = 0.93; and CFI = 0.93. The results indicated that the model fit was at the expected level (Figure 1).

### 4.4 Internal consistency and stability of the questionnaire

Table 2 shows the reliability of the 28‐item instrument. The highest Cronbach's alpha was reported as being 0.89. The test–retest reliability approach was conducted on 100 student participants once, just after their main examination and within two weeks after the examination. The intraclass correlation coefficient (ICC) for the questionnaire was reported to be 0.92 and ranged from 0.81–0.98 for sub‐factors.

## 5 DISCUSSION

There are many studies conducted on nurses’ professionalization. To conduct such studies efficiently, reliable and valid instruments (e.g. questionnaire) are essential. Therefore, designing, revising and validating a questionnaire to evaluate nurses’ professionalization are of great importance for the research community, especially nursing researchers. In this sense, a specific questionnaire concerning nursing students’ professionalization (IPASN‐P), including 28 items in eight dimensions, was translated into Persian and then the validation conditions were determined. This questionnaire is quite easy to complete and medical staff and nursing students in nursing universities can complete it in less than 15 min. The translation process was carried by experts, following translation principles and paying accurate attention to the cultural semantic matching between the two languages. Therefore, the translation and validation processes comply with the main existing guidelines (Eghbali et al., 2020; Polit & Yang, 2015).

To validate the questionnaire, face validity (qualitative), content validity (qualitative), construct validity (exploratory factor analysis and confirmatory factor analysis) and internal consistency (Cronbach's alpha) were sought. The face validation was approved by asking students to carry out a qualitative analysis. Then, experts were interviewed and the content validity and cultural matching were ensured.

The instrument development process normally begins with EFA and factor structure. Then, the resulting model is verified using a CFA. In the present study, the EFA resulted in an 8‐factor model of 28 items for the instrument and the model was confirmed by a CFA. A major reason for using an EFA is to generate a theory about the essential structures of an instrument and this process is followed by a CFA, which should be performed using data other than those used in the EFA. Therefore, in the present study, the CFA was conducted with a new set of data collected from a sample consisting of 300 subjects (Hung et al., 2010). During the CFA, the goodness of fit was calculated using several variables. The most frequently used indices include NFI, NNFI, AGFI, GFI, RMSEA and CFI, all of which were relatively acceptable.

One of the indices which are commonly used in the evaluation of the convenience of a model is the chi‐square (χ2). The chi‐square value and degree of freedom (*df*) for a model could provide normed fit value (χ2/*df*) where values less than 3.0 indicate a good model fit (Kline, 1998). However, this index is sensitive to sample size and when sample size is more than 200 it is not a valuable index for model fit (Lerman et al., 2010; Limbers et al., 2009). Thus in current study, since sample size was 300, normed fit was not used.

Cronbach's alpha indices were indicative of high internal consistency among the items of the questionnaire; hence, the reliability of the nursing students’ professionalization attitude was approved. In a similar study, Hisar (Hisar et al., 2010) and Xiao (Xiao et al., 2017) reported a reliability index between 0.71 to 0.84–0.67 to 0.89, respectively. Therefore, the results of this study are in line with that of Hisar's and Xiao in accrediting the total reliability of the questionnaire. However, one should be noticed that using scree plot and eigenvalues are traditional methods and instead it is recommended to use a newer approach such as parallel analysis in the future studies.

### 5.1 Limitations

Although this study benefited from a good sample size and it contributes to the necessity of practice development and educational needs of pre‐licensure nursing students, a few limitations should be acknowledged. Firstly, the sample was recruited from a limited number of nursing faculties. Secondly, we used the English version of the questionnaire for forward–backward translation. Perhaps not using the original Turkish version of the question might be a limitation.

[Correction added on 5 January 2021 after first online publication: The preceding sentence has been amended from ‘Perhaps using the…’ to ‘Perhaps not using the…’.]

## 6 CONCLUSION

The study findings suggest that the Persian version of 28‐item IPASN questionnaire (IPASN‐P) is a reliable and valid instrument. Indeed, the questionnaire is worthy of use and further helps to determine the professional attitudes of student nurses.

## 7 CONSENT TO PUBLISH

Not applicable.

## CONFLICT OF INTEREST

The authors declare that they have no competing interests.

## AUTHOR’S CONTRIBUTIONS

MHK was the study supervisor and contributed to all aspect of the study. ME and FGH collected data. RB was the main investigator and provided the first draft. AM was the study advisor and contributed to the study design, critically reviewed the paper and provided the final draft. RB was the study advisor and contributed to the writing process, and the statistical advisor and contributed to data analysis. All authors read and approved of the final manuscript.

## ETHICAL APPROVAL

The University Of Social Welfare and Rehabilitation Sciences Ethics Committee approved the study. All participants signed informed consent form.

## Data Availability

The data sets are available from the corresponding author on request.
